# Perceived discrimination as a mediator between cultural identity and mental health symptoms among racial/ethnic minority adults in the United States: insights from the Health Information National Trends Survey 6

**DOI:** 10.3389/fpsyg.2024.1233919

**Published:** 2024-02-28

**Authors:** Lulin Zhou, Jonathan Aseye Nutakor, Ebenezer Larnyo, Stephen Addai-Dansoh, Yupeng Cui, Nutifafa Eugene Yaw Dey

**Affiliations:** ^1^School of Management, Jiangsu University, Zhenjiang, Jiangsu Province, China; ^2^Center for Black Studies Research, University of California, Santa Barbara, Santa Barbara, CA, United States; ^3^Department of Psychology, University of Ghana, Accra, Ghana

**Keywords:** cultural identity, perceived discrimination, mental health symptoms, racial/ethnic minority adults, socioeconomic factors

## Abstract

**Objective:**

This study examined the role of perceived discrimination as a mediator between cultural identity and mental health symptoms among adults from racial/ethnic minority groups in the United States.

**Methods:**

Data were gathered from the National Cancer Institute’s (NCI) Health Information National Trends Survey (HINTS) 6, a nationally representative survey. The mediating role of perceived discrimination was investigated using structural equation modeling (SEM) analysis.

**Results:**

The study found significant associations between demographic and lifestyle factors and mental health symptoms. Non-Hispanic Blacks or African Americans and Hispanics were less likely than Non-Hispanic Whites to have mental health symptoms. Individuals between the ages of 35 and 49, 50 to 64, 65 to 74, and 75 and older had lower odds of mental health symptoms. Gender differences revealed that females had a higher risk of mental health issues than males. Socioeconomic factors, such as household income and employment status, played a significant role, with higher household income and employment status being associated with a decreased likelihood of mental health symptoms. The study emphasizes the role of perceived discrimination as a mediator, suggesting that it fully mediated the association between cultural identity and mental health symptoms. These findings highlight the significance of addressing discrimination experiences in fostering the mental health of adults from diverse backgrounds.

**Conclusion:**

The findings highlight the need to take racial, ethnic, and socioeconomic inequities, as well as cultural identity and prejudice, into account in mental health research and interventions. The identified risk and protective factors can guide interventions and policies to enhance this population’s mental health.

## 1 Introduction

In the United States, mental health symptoms among racial/ethnic minority populations have garnered increasing attention due to their significant impact on individual well-being and public health (Mongelli et al., 2020). The intricate interplay between cultural identity, perceived discrimination, and mental health outcomes highlights the need for exhaustive research into the underlying mechanisms contributing to mental health disparities (Adams and Miller, 2022). Using data from the Health Information National Trends Survey (HINTS) 6 (National Cancer Institute, 2023), this study seeks to investigate the mediating role of perceived discrimination in the relationship between cultural identity and mental health symptoms among racial/ethnic minority adults in the United States.

Mental health disorders affect millions worldwide, with significant regional and cultural differences (Liu et al., 2020; Nutakor et al., 2021). The prevalence and impact of mental health disorders have prompted international organizations and governments to make concerted efforts to address the problem (Kopinak, 2015; Nutakor et al., 2023a,b,c). Despite these efforts, deviations from global targets persist, especially among populations of racial/ethnic minorities (Mongelli et al., 2020). Recent studies have highlighted the importance of understanding the factors contributing to disparities in mental health among minority adults. Adams and Miller (2022) discovered disparities in mental health outcomes among racial/ethnic minority populations, highlighting the need for targeted interventions and research to address these disparities. These findings highlight the significance of investigating the intricate relationships between cultural identity, perceived discrimination, and mental health symptoms.

Numerous attempts have been made by state and non-state actors in the United States to address mental health disparities among racial/ethnic minority populations (American Public Health Association, 2018). Policies, programs, and actions have been implemented to enhance mental health and prevent discrimination (American Public Health Association, 2018). For instance, the National Alliance on Mental Illness (NAMI) has advocated for culturally sensitive mental health services to meet the unique requirements of minority populations (Watts et al., 2023). Additionally, initiatives such as the Minority Mental Health Programme of the Substance Abuse and Mental Health Services Administration (SAMHSA) seek to improve access to quality mental health care and reduce disparities (Yang et al., 2020). These efforts are consistent with the global agenda outlined in the Mental Health Action Plan of the World Health Organization, which emphasizes the importance of equity and inclusion in mental health services (WHO, 2022). In a research conducted by Schwartz and Jahn (2020), the effectiveness of regions in addressing mental health inequalities among racial/ethnic minority groups was ranked. This ranking provides insights into the effectiveness of various interventions and policies implemented by various regions, providing valuable teachings for enhancing mental health outcomes. Understanding these regional differences may inform the design and implementation of targeted strategies, considering the successful approaches employed in high-performing regions.

Relevant and current literature highlights gaps in methods, data, analytical approaches, and study areas about the mediating role of perceived discrimination in the association between cultural identity and mental health symptoms among racial/ethnic minority adults (Vines et al., 2017; Woo et al., 2019; Eylem et al., 2020; Vargas et al., 2020). While previous research has investigated the individual associations between cultural identity, perceived discrimination, and mental health outcomes, there is a dearth of research examining their comprehensive interrelationships. In addition, methodological and analytical approaches must consider the complexity of these relationships. This study seeks to address these gaps by utilizing the nationally representative HINTS 6 dataset, which provides a robust platform for investigating the role of perceived discrimination as a mediator.

Understanding the challenges faced by racial and ethnic minority populations in the United States requires examining the relationship between cultural identity, mental health, and perceived discrimination. Cultural identity plays a significant role in an individual’s psychological well-being and resilience. However, perceived discrimination can have serious detrimental effects on mental health. A study by Ricci et al. (2023) highlights the negative impact of racism and discrimination on ethnic minorities’ health, underscoring the need for anti-racism policies and the importance of embracing pluralistic societies to address mental health disparities. In addition, the research conducted by Huey et al. (2023) has shown that Cognitive Behavioral Therapy (CBT) is generally effective. However, the efficacy of CBT might be lower for ethnic minorities. This suggests that there is a need for cultural adaptations and sensitivity training for therapists. These studies highlight the significance of incorporating cultural sensitivity and anti-racism frameworks into mental health interventions. Ensuring that these interventions are effective and equitable for all communities, especially those most vulnerable to the harmful effects of discrimination, is crucial.

Furthermore, while the main focus of this study is to examine the role of perceived discrimination in mediating the relationship between cultural identity and mental health symptoms among adults from racial and ethnic minority groups, it is also essential to analyze the demographic variables of the participants for several reasons. Firstly, understanding the demographic makeup of the sample population is crucial in interpreting the study findings in a broader societal context. Factors such as age, gender, race, ethnicity, and socioeconomic status can significantly impact individuals’ experiences, perceptions, and responses. Secondly, analyzing the demographic variables helps identify potential disparities or patterns within different subgroups, highlighting areas where targeted interventions or policies may be necessary to address inequities. By examining the participant demographics alongside the study outcomes, we aim to provide a comprehensive understanding of the complex dynamics involved and ensure that our findings are relevant and applicable to diverse populations.

This study’s primary objective is to examine the role of perceived discrimination as a mediator in the association between cultural identity and mental health symptoms among racial/ethnic minority adults in the United States. The HINTS 6 dataset, which offers a diverse and representative sample, will be used to investigate these complex associations. Statistical analyses, such as structural equation modelling (SEM), will be used to investigate the fundamental mechanisms and pathways. The findings of this study will provide evidence for the development of targeted interventions aimed at reducing mental health disparities and promoting the well-being of racial/ethnic minority populations, which have significant implications for policy design and implementation. Understanding the mediating role of perceived discrimination enables policymakers to implement strategies to combat discrimination, increase cultural inclusivity, and ultimately improve mental health outcomes among racial/ethnic minority adults in the United States.

## 2 Materials and methods

### 2.1 Participants

The Health Information National Trends Survey (HINTS) (National Cancer Institute, 2023), a nationally representative survey conducted by the National Cancer Institute (NCI) since 2003, provided the data for this study. HINTS offers valuable insights into the American public’s knowledge, attitudes, and use of cancer- and health-related information to enhance health communication strategies across diverse populations. For this study, the HINTS 6 survey information was utilized. The HINTS 6 survey targeted 18-year-old or older non-institutionalized civilians residing in the United States. The strategy for sampling consisted of a two-stage plan. In the initial phase, a stratified sample of residential addresses was selected, considering both rural and urban areas and areas with high and low concentrations of minority adult populations. The addresses were randomly selected from a database maintained by Marketing Systems Group (MSG) that contains all non-vacant residential addresses in the United States, including P.O. boxes and seasonal addresses. The sampling frame was divided into four explicit sampling strata: (1) urban areas with high concentrations of minority adults; (2) urban areas with low concentrations of minority adults; (3) rural areas with high concentrations of minority adults; and (4) rural areas with low concentrations of minority adults. This stratification made it possible to oversample the high-minority and rural strata to enhance estimates for these subpopulations. Within each stratum, an equal-probability sample of addresses was chosen, totaling 29,600 addresses for HINTS 6. The compilation of data for HINTS 6 occurred between March 7 and November 8, 2022. A modified Dillman approach was used for the mailing protocol, which consisted of four mailings to selected households: the initial mailing, a reminder postcard, and two subsequent mailings. A subsample of nonresponders were sent a third follow-up mailing in response to a lower-than-anticipated response rate.

### 2.2 Measures

The 4-item Patient Health Questionnaire for Depression and Anxiety (PHQ-4), a combination of the Patient Health Questionnaire-2 (PHQ-2) and the Generalized Anxiety Disorder-2 (GAD-2) scales, was adopted to measure the psychological distress of respondents (Huang et al., 2023). For the PHQ-2, respondents were asked, “Over the past 2 weeks, how often have you been bothered by the following: a little interest or pleasure in doing things, feeling down, depressed, or hopelessness?” Each item is scored on a 4-point Likert scale ranging from 0 (nearly every day) to 3 (not at all). Similarly, for GAD-2, respondents were asked, “Over the past 2 weeks, how often have you been bothered by: Feeling nervous, anxious, or on edge and unable to stop or control worrying?” The total PHQ-4 score can range between 0 and 12, and the total PHQ-2 and GAD-2 scores can range between 0 and 6. Higher scores indicate higher depression and anxiety levels. Based on the total score (0–12), psychological distress is classified as none (0–2), mild (3–5), moderate (6–9), or severe (10–12). A score of 3 or higher indicated at least mild anxiety and depression symptoms. All four items served as observed variables for the latent construct of mental health symptoms in structural equation modeling (SEM) analyses.

Race or ethnicity was used to assess cultural identity. Respondents were requested to select their race from the options: Non-Hispanic White, Non-Hispanic Black or African American, Hispanic, Non-Hispanic Asian, and Non-Hispanic Other. Respondents were asked if they had ever received unfair treatment or discrimination in medical care because of their race or ethnicity, with a yes or no response choice, in order to measure perceived discrimination. To ensure that the measures for cultural identity and perceived discrimination are valid, we took additional steps. Regarding cultural identity, we followed established demographic research practices and used race or ethnicity as the basis for classification. This provided a clear framework for assessing how cultural identity influences mental health. Regarding perceived discrimination, we used a binary choice approach that effectively captures instances of unfair treatment linked to race or ethnicity. This is important for understanding the mental health implications of such experiences. The U.S. Census Bureau has conducted extensive research to improve data on race and ethnicity, and we used their approach for our study. This research emphasizes the evolution of race and ethnicity classification, ensuring that data collection methods remain relevant and accurately reflect the diversity of the American population (United States Census Bureau, 2017).

Age, gender, marital status, education, household income, occupation, smoking status, alcohol status, physical activity, and body mass index (BMI) were considered sociodemographic variables. Age was classified into five ranges, gender as male or female, and marital status as married, divorced/separated, widowed, or single/never married. Based on educational attainment, the level of education was divided into five categories and household income was divided into five quintiles. The occupation classifications included employed, homemaker, student, retired, disabled, multiple occupation status, unemployed for 1 year or more, unemployed for less than 1 year, and other occupation. Current, former, or non-smokers were classified according to their smoking status. Alcohol consumption was categorized as never or currently, and BMI as underweight, normal weight, overweight, or obese.

### 2.3 Statistical analysis

STATA SE version 14.2 (Stata Corp, College Station, TX) and Intellectus Statistics were used to analyze the data for this study (Nutakor et al., 2020). A descriptive statistical analysis was conducted to provide a summary of the relevant variables. This analysis entailed a summary of the variables’ distributional characteristics. A binary logistic regression was conducted to investigate the association between the variables of interest and mental health symptoms. This regression analysis aimed to determine the relationship between the independent variable (cultural identity) and the dependent variable (mental health symptoms). In addition, structural equation modelling (SEM) was used to investigate the role of perceived discrimination as a mediator in the relationship between cultural identity and mental health symptoms. SEM is a statistical method that permits the simultaneous examination of complex relationships between multiple variables. The following regression formula was used for this analysis: Mental Health Symptoms = β0 + β1(Cultural Identity) + β2(Perceived Discrimination) + β3(Cultural Identity * Perceived Discrimination) + ε. The intercept β0 represents the expected value of the dependent variable when all other variables are zero, and ε represents the error term accounting for unexplained variation in mental health symptoms not captured by the independent variables. This study sought to understand the relationships between cultural identity, perceived discrimination, and mental health symptoms by employing these statistical methods. The application of rigorous statistical analyses permitted the examination of direct and mediated effects, yielding valuable insights into the underlying mechanisms at play.

## 3 Results

The study’s results revealed intriguing patterns in demographic characteristics across various racial and ethnic groups (Table 1). The highest proportion of non-Hispanic Whites was identified in the age range of 50 to 64, whereas the highest proportion of non-Hispanic Asians was discovered in the age range of 18 to 34. While Non-Hispanic Blacks or African Americans were predominately male, Non-Hispanic Whites were predominately female. Additionally, the majority of non-Hispanic Whites were married, compared to the majority of single or never married Hispanics. Education levels vary among racial/ethnic groups, with Non-Hispanic Whites and Non-Hispanic Asians having the most significant percentages of Bachelor’s and post-Baccalaureate degree holders, respectively. The majority of non-Hispanic Whites reported household incomes of $75,000 or more, whereas the majority of Hispanics reported household incomes of under $20,000. Most Non-Hispanic Whites were employed, compared to the majority of Non-Hispanic Blacks or African Americans who were retired. The majority of Non-Hispanic Whites had never smoked and were current drinkers, while the majority of Hispanics were current smokers and had never smoked. The majority of Non-Hispanic Whites were inactive, whereas the majority of Hispanics were physically active. In comparing body mass index (BMI) categories across racial/ethnic groups, most Non-Hispanic Whites fell into the normal overweight category. On the other hand, most Non-Hispanic Blacks or African Americans fell into the obese category.

**Table 1:** Summary statistics of study variables.

Variable	*N* = 4,762 n (%)	Non-Hispanic White *n* = 2,817	Non-Hispanic Black or African American *n* = 722	Hispanic *n* = 809	Non-Hispanic Asian *n* = 254	Non-Hispanic Other *n* = 160
*Age*						
18–34	763 (16.02)	345 (12.25)	101 (13.99)	208 (25.71)	75 (29.53)	34 (21.25)
35–49	1,032 (21.67)	536 (19.03)	149 (20.64)	230 (28.43)	78 (30.71)	39 (24.38)
50–64	1,385 (29.08)	822 (29.18)	249 (34.49)	211 (26.08)	55 (21.65)	48 (30.00)
65–74	1,014 (21.29)	677 (24.03)	168 (23.27)	112 (13.84)	31 (12.20)	26 (16.25)
75 +	568 (11.93)	437 (15.51)	55 (7.62)	48 (5.93)	15 (5.91)	13 (8.12)
*Gender*						
Male	1937 (40.68)	1,206 (42.81)	233 (32.27)	311 (38.44)	129 (50.79)	58 (36.25)
Female	2,825 (59.32)	1,611 (57.19)	489 (67.73)	498 (61.56)	125 (49.21)	102 (63.75)
*Marital status*						
Married	2,557 (53.70)	1,627 (57.76)	249 (34.49)	443 (54.76)	161 (63.39)	77 (48.12)
Divorced/Separated	836 (17.56)	467 (16.58)	156 (21.61)	161 (19.90)	14 (5.51)	38 (23.75)
Widowed	433 (9.09)	296 (10.51)	72 (9.97)	37 (4.57)	16 (6.30)	12 (7.50)
Single/never been married	936 (19.66)	427 (15.16)	245 (33.93)	168 (20.77)	63 (24.80)	33 (20.62)
*Education*						
Less than High school	248 (5.21)	86 (3.05)	48 (6.65)	95 (11.74)	11 (4.33)	8 (5.00)
High school graduate	784 (16.46)	451 (16.01)	118 (16.34)	170 (21.01)	21 (8.27)	24 (15.00)
Some College	1,358 (28.52)	741 (26.30)	275 (38.09)	247 (30.53)	39 (15.35)	56 (35.00)
Bachelor’s degree	1,396 (29.32)	870 (30.88)	179 (24.79)	193 (23.86)	110 (43.31)	44 (27.50)
Post - Baccalaureate degree	976 (20.50)	669 (23.75)	102 (14.13)	104 (12.86)	73 (28.74)	28 (17.50)
*Household income*						
Less than $20,000	703 (14.76)	288 (10.22)	181 (25.07)	169 (20.89)	34 (13.39)	31 (19.38)
$20,000 to < $35,000	602 (12.64)	313 (11.11)	115 (15.93)	132 (16.32)	19 (7.48)	23 (14.37)
$35,000 to < $50,000	617 (12.96)	353 (12.53)	102 (14.13)	120 (14.83)	21 (8.27)	21 (13.12)
$50,000 to < $75,000	840 (17.64)	496 (17.61)	131 (18.14)	145 (17.92)	43 (16.93)	25 (15.62)
$75,000 or more	2000 (42.00)	1,367 (48.53)	193 (26.73)	243 (30.04)	137 (53.94)	60 (37.50)
*Occupation*						
Employed only	2,424 (50.90)	1,397 (49.59)	353 (48.89)	432 (53.40)	162 (63.78)	80 (50.00)
Homemaker only	165 (3.46)	86 (3.05)	10 (1.39)	53 (6.55)	10 (3.94)	6 (3.75)
Student only	54 (1.13)	22 (0.78)	9 (1.25)	8 (0.99)	9 (3.54)	6 (3.75)
Retired only	1,290 (27.09)	923 (32.77)	182 (25.21)	121 (14.96)	36 (14.17)	28 (17.50)
Disabled only	212 (4.45)	109 (3.87)	53 (7.34)	38 (4.70)	1 (0.39)	11 (6.88)
Multiple Occupation statuses selected	384 (8.06)	181 (6.43)	74 (10.25)	91 (11.25)	19 (7.48)	19 (11.88)
Unemployed for 1 year or more only	128 (2.69)	48 (1.70)	22 (3.05)	38 (4.70)	13 (5.12)	7 (4.38)
Unemployed for less than 1 year only	78 (1.64)	34 (1.21)	15 (2.08)	24 (2.97)	3 (1.18)	2 (1.25)
Other Occupation only	27 (0.57)	17 (0.60)	4 (0.55)	4 (0.49)	1 (0.39)	1 (0.62)
*Smoking status*						
Current	496 (10.42)	300 (10.65)	102 (14.13)	55 (6.80)	14 (5.51)	25 (15.62)
Former	1,192 (25.03)	856 (30.39)	133 (18.42)	139 (17.18)	24 (9.45)	40 (25.00)
Never	3,074 (64.55)	1,661 (58.96)	487 (67.45)	615 (76.02)	216 (85.04)	95 (59.38)
*Drinking status*						
Never	2,446 (51.36)	1,327 (47.11)	421 (58.31)	438 (54.14)	161 (63.39)	99 (61.88)
Current	2,316 (48.64)	1,490 (52.89)	301 (41.69)	371 (45.86)	93 (36.61)	61 (38.12)
*Physical activity*						
Inactive	2,969 (62.35)	1,658 (58.86)	514 (71.19)	531 (65.64)	158 (62.20)	108 (67.50)
Active	1793 (37.65)	1,159 (41.14)	208 (28.81)	278 (34.36)	96 (37.80)	52 (32.50)
*Body Mass Index (BMI)*						
Underweight	53 (1.11)	39 (1.38)	6 (0.83)	1 (0.12)	4 (1.57)	3 (1.88)
Normal weight	1,395 (29.29)	894 (31.74)	139 (19.25)	181 (22.37)	137 (53.94)	44 (27.50)
Overweight	1,594 (33.47)	947 (33.62)	242 (33.52)	279 (34.49)	83 (32.68)	43 (26.88)
Obese	1720 (36.12)	937 (33.26)	335 (46.40)	348 (43.02)	30 (11.81)	70 (43.75)

**Figure 1:**
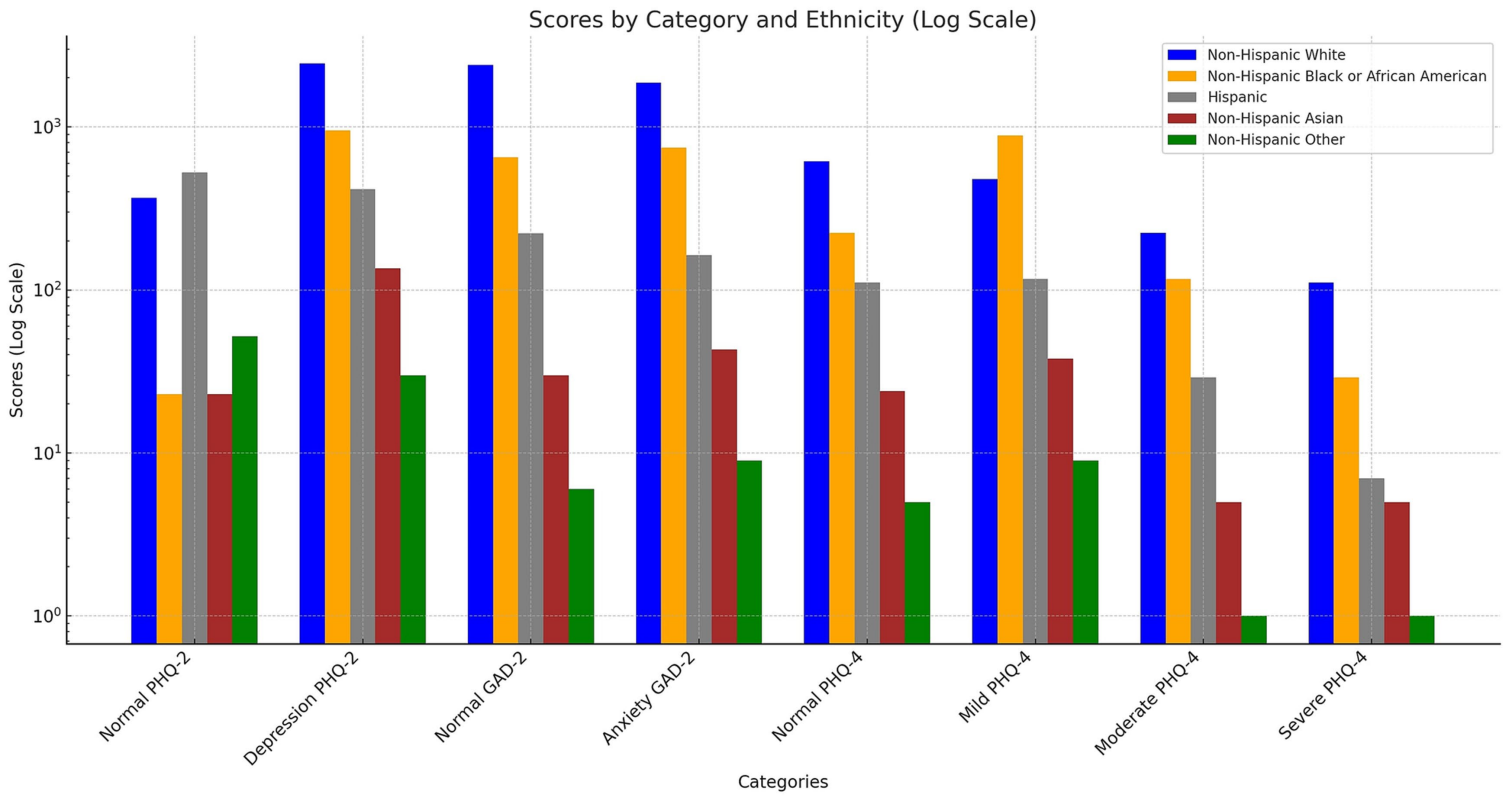
The distribution of mental health outcomes across various racial/ethnic groups.

Figure 1 compares mental health assessments of various ethnicities using a logarithmic scale to represent a broad range of scores from tools that measure depression and anxiety, such as PHQ and GAD. The scores of Non-Hispanic whites are higher, indicating fewer symptoms, especially in the ‘Normal’ range, while they are lower for severe symptoms, implying better mental health overall. In contrast, Non-Hispanic Black or African American individuals show higher scores in the ‘Severe PHQ-4’ category, which suggests a higher prevalence of severe mental health conditions. On the other hand, Hispanic scores are generally lower across the board, which could be due to underreporting or limited access to mental health services. The chart uses different colors to represent each group - blue for Non-Hispanic White, orange for Non-Hispanic Black or African American, grey for Hispanic, brown for Non-Hispanic Asian, and green for Non-Hispanic Other - making it easy to distinguish between them.

**Figure 2:**
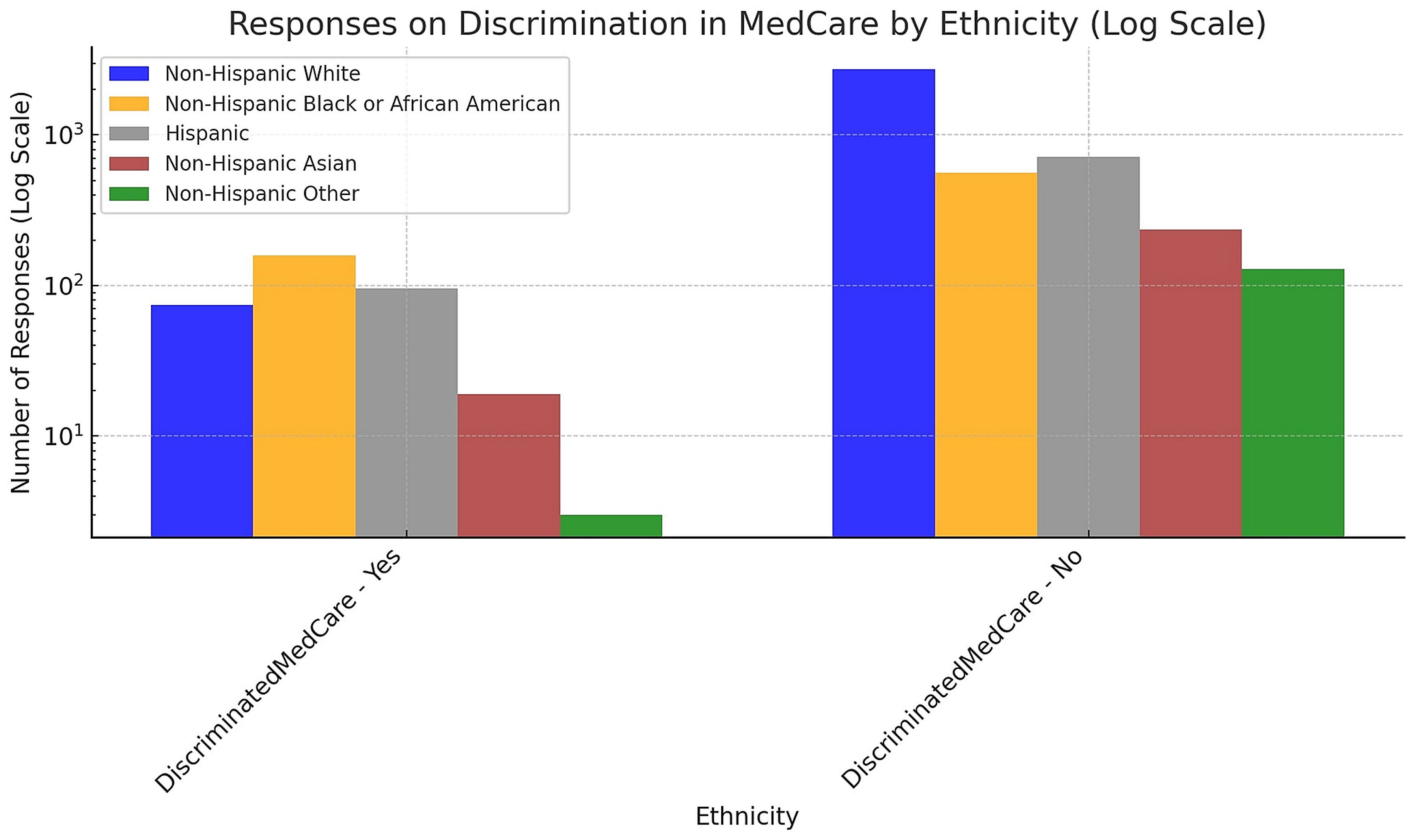
The prevalence of experienced discrimination in medical care among various racial/ethnic groups.

The data in Figure 2 displays data on reported experiences of discrimination in medical care among various ethnic groups, measured on a logarithmic scale to accommodate a wide range of responses. The chart shows a significant difference in the reported experiences of discrimination between those who responded ‘Yes’ to experiencing discrimination and those who answered ‘No’. The Non-Hispanic White group, indicated by the blue bars, has a substantial number of responses denying discrimination in MedCare, with a considerably lower number reporting discrimination. This trend is consistent across all ethnic groups, with the number of ‘No’ responses exceeding ‘Yes’ responses by a significant margin. However, each ethnic group has a non-negligible fraction of individuals who report experiencing discrimination. Among the ethnic groups, Non-Hispanic Black or African American respondents, represented by the orange bars, have a relatively higher count than Non-Hispanic Asians and Non-Hispanic Others, as evidenced by the height of the bars corresponding to a ‘Yes’ response.

Table 2 displays the results of a binary logistic regression analysis that examined the probabilities of mental health symptoms concerning various demographic and lifestyle factors. Non-Hispanic Blacks or African Americans have 36% lower odds of mental health symptoms than Non-Hispanic Whites [OR = 0.64, 95% CI: (0.53–0.77), *p* < 0.001], while Hispanics have 27% lower odds [OR = 0.73, 95% CI: (0.60–0.87), *p* < 0.001]. Regarding age groups, individuals aged 35–49 have 41% lower odds of mental health symptoms [OR = 0.59, 95% CI: (0.47–0.74), *p* < 0.001], those aged 50–64 have 63% lower odds of mental health symptoms [OR = 0.37, 95% CI: (0.29–0.46), *p* < 0.001], individuals aged 65–74 have 72% lower odds of mental health symptoms [OR = 0.28, 95% CI: (0.21–0.37), *p* < 0.001], and individuals aged 75 and older have 71% lower odds of mental health symptoms [OR = 0.29, 95% CI: (0.21–0.41), *p* < 0.001]. Females have a 29% higher risk of mental health issues than males [OR = 1.29, 95% CI: (1.13–1.46), *p* < 0.001]. Individuals who have never been married or have never been married have a 20% increased risk of mental health symptoms [OR = 1.20, 95% CI: (1.00–1.44), *p* < 0.043]. Individuals with a household income between $35,000 and less than $50,000 have 24% lower odds of mental health symptoms [OR = 0.76, 95% CI: (0.59–0.98), *p* < 0.041], whereas those with a household income of $75,000 or more have 37% lower odds [OR = 0.63, 95% CI: (0.49–0.80), *p* < 0.001]. When compared to those who are employed, disabled individuals have a 79% higher risk of mental health symptoms [OR = 1.79, 95% CI: (1.25–2.55), *p* < 0.001], and individuals with multiple occupation statuses have a 37% higher risk of mental health symptoms [OR = 1.37, 95% CI: (1.07–1.75), *p* < 0.012]. Furthermore, individuals who have been unemployed for a year or more had a 127% increased risk of developing mental health problems [OR = 2.27, 95% CI: (1.42–3.61), *p* < 0.001]. Taking lifestyle factors into account, former smokers have 30% lower odds of mental health symptoms than current smokers [OR = 0.70, 95% CI: (0.55–0.90), *p* < 0.005], and individuals who have never smoked have 46% lower odds [OR = 0.54, 95% CI: (0.43–0.68), *p* < 0.001]. In addition, compared to those who have never consumed alcohol, current drinkers have a 35% increased risk of mental health symptoms [OR = 1.35, 95% CI: (1.19–1.54), *p* < 0.001]. Finally, levels of physical activity were associated with mental health symptoms. Physically active individuals have a 33% lower risk of mental health symptoms than inactive individuals [OR = 0.67, 95% CI: (0.59–0.76), *p* < 0.001].

**Table 2:** Binary logistic regression analysis examining the probabilities of mental health disorders concerning various demographic and lifestyle factors.

	Odds Ratio	95% CI	*p* - value
*Cultural identity*			
Non-Hispanic White	ref		
Non-Hispanic Black or African American	0.64	[0.53–0.77]	0.001
Hispanic	0.73	[0.60–0.87]	0.001
Non-Hispanic Asian	0.97	[0.72–1.28]	0.810
Non-Hispanic Other	0.77	[0.54–1.09]	0.142
*Perceived discrimination*			
Yes	ref		
No	0.66	[0.52–0.85]	0.001
*Age*			
18–34	ref		
35–49	0.59	[0.47–0.74]	0.001
50–64	0.37	[0.29–0.46]	0.001
65–74	0.28	[0.21–0.37]	0.001
75 +	0.29	[0.21–0.41]	0.001
*Gender*			
Male	ref		
Female	1.29	[1.13–1.46]	0.001
*Marital status*			
Married	ref		
Divorced/Separated	1.12	[0.94–1.35]	0.187
Widowed	1.13	[0.89–1.43]	0.301
Single/never been married	1.20	[1.00–1.44]	0.043
*Education*			
Less than High school	ref		
High school graduate	0.96	[0.70–1.32]	0.830
Some College	1.04	[0.77–1.42]	0.770
Bachelor’s degree	1.05	[0.77–1.44]	0.733
Post - Baccalaureate degree	1.14	[0.82–1.59]	0.425
*Household income*			
Less than $20,000	ref		
$20,000 to < $35,000	0.80	[0.63–1.03]	0.086
$35,000 to < $50,000	0.76	[0.59–0.98]	0.041
$50,000 to < $75,000	0.78	[0.61–1.00]	0.051
$75,000 or more	0.63	[0.49–0.80]	0.001
*Occupation*			
Employed only	ref		
Homemaker only	0.96	[0.67–1.35]	0.806
Student only	1.39	[0.70–2.74]	0.335
Retired only	0.86	[0.69–1.05]	0.145
Disabled only	1.79	[1.25–2.55]	0.001
Multiple Occupation statuses selected	1.37	[1.07–1.75]	0.012
Unemployed for 1 year or more only	2.27	[1.42–3.61]	0.001
Unemployed for less than 1 year only	1.57	[0.92–2.66]	0.093
Other Occupation only	0.78	[0.35–1.74]	0.550
*Smoking status*			
Current	ref		
Former	0.70	[0.55–0.90]	0.005
Never	0.54	[0.43–0.68]	0.001
*Drinking status*			
Never	ref		
Current	1.35	[1.19–1.54]	0.001
*Physical activity*			
Inactive	ref		
Active	0.67	[0.59–0.76]	0.001
*Body Mass Index (BMI)*			
Underweight	ref		
Normal weight	0.84	[0.46–1.56]	0.601
Overweight	0.81	[0.44–1.49]	0.508
Obese	1.01	[0.55–1.87]	0.957

OR, Odds Ratio; CI, Confidence Intervals.

**Figure 3:**
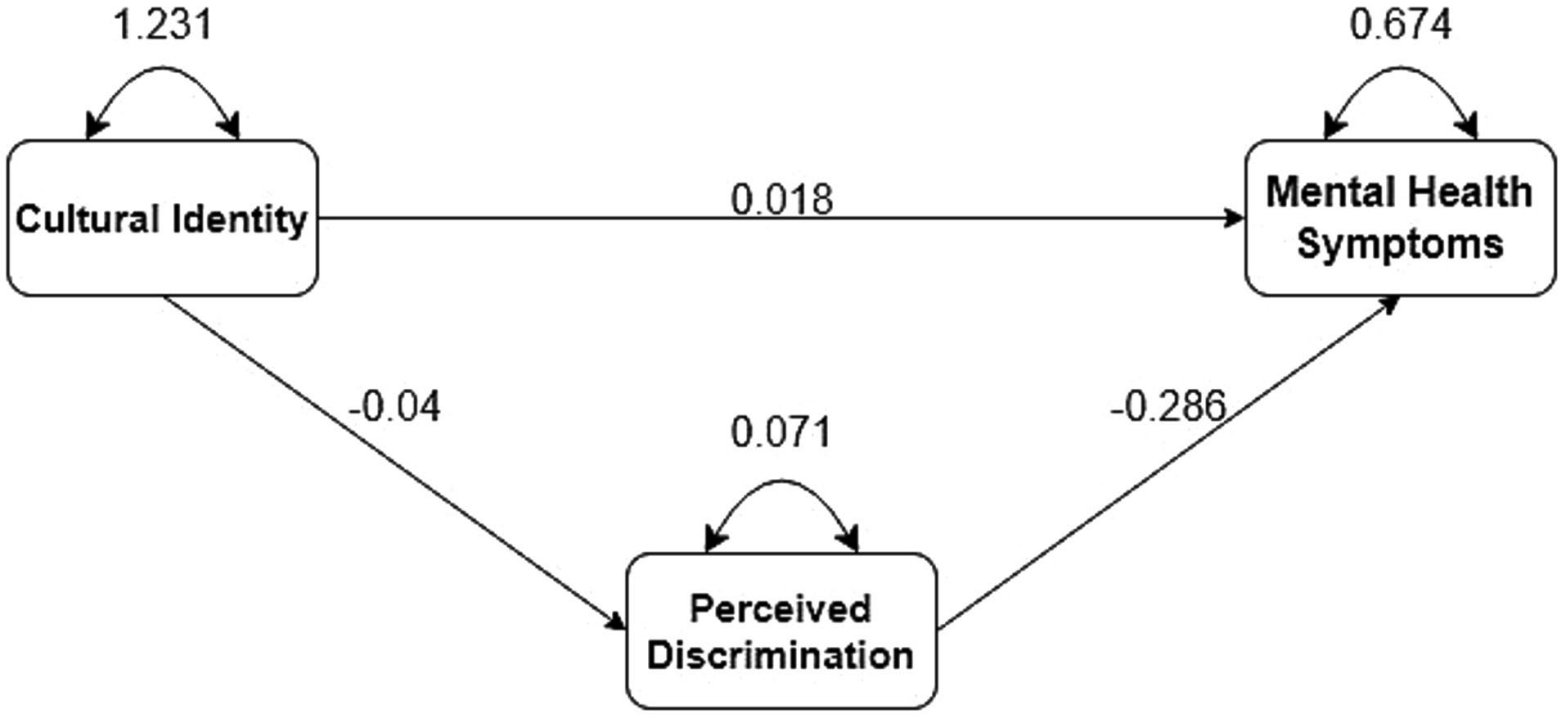
Node diagram for the path analysis model.

The results of the mediation analysis examining the relationship between cultural identity, perceived discrimination, and mental health symptoms are presented in Table 3. With a significance level of α = 0.05, a mediation test was conducted to determine whether perceived discrimination mediated the relationship between cultural identity and mental health symptoms. The direct relationship between cultural identity and mental health symptoms was not shown to be significant, suggesting that experienced discrimination may act as a full mediator. Following established methods (Preacher and Hayes, 2004; Zhao et al., 2010; Gunzler et al., 2013), the indirect and total effects of perceived discrimination on the association between cultural identity and mental health symptoms were investigated to assess full mediation. The results demonstrated that the indirect effect of perceived discrimination on the association between cultural identity and mental health symptoms was statistically significant (B = 0.01, *z* = 5.59, *p* < 0.001). This suggests that a one-unit increase in cultural identity, as measured by its effect on perceived discrimination, is associated with a 0.01-unit increase in mental health symptoms. In addition, the total effect of cultural identity on mental health symptoms was significant (B = 0.03, *z* = 2.73, *p* < 0.006), indicating that a one-unit increase in cultural identity is associated with a 0.03-unit increase in mental health symptoms. Given the significance of both the indirect and total effects, the findings support full mediation by perceived discrimination (Preacher and Hayes, 2004; Zhao et al., 2010; Gunzler et al., 2013). The node diagram for the path analysis model is shown in Figure 3.

**Table 3:** Unstandardized loadings (standard errors), standardized loadings, and significance levels for each parameter in the path analysis model.

Parameter Estimate	Unstandardized	Standardized	*p*
Regressions			
Cultural identity → Mental health	0.02 (0.01)	0.02	0.097
Cultural identity → Perceived discrimination	−0.04 (0.003)	−0.16	< 0.001
Perceived discrimination → Mental health	−0.29 (0.04)	−0.09	< 0.001
Indirect effect of mental health on cultural identity by perceived discrimination	0.01 (0.002)	0.02	< 0.001
Total effect of mental health on cultural identity	0.03 (0.01)	0.04	0.006
Errors			
Error in cultural identity	1.23 (0.03)	1	< 0.001
Error in perceived discrimination	0.07 (0.001)	0.97	< 0.001
Error in mental health	0.67 (0.01)	0.99	< 0.001

p, Significance level.

## 4 Discussion

The binary logistic regression analysis results presented in Table 2 provide significant associations between demographic and lifestyle factors and adult mental health symptoms. These findings have significant implications for understanding the mental health outcomes of this population and may influence the development of targeted interventions and policies.

According to the study, non-Hispanic Blacks or African Americans, and Hispanics have significantly lower odds of mental health symptoms than non-Hispanic Whites. This suggests a positive association between minority racial and ethnic group membership and adult mental health outcomes. Recent studies conducted by Weaver et al. (2015) and Ettman et al. (2020) also observed lower rates of mental health symptoms among minority populations, confirming the results of this study. These findings highlight the significance of addressing disparities and providing culturally sensitive mental health services to adult populations from different cultural backgrounds. Regarding age groups, our research revealed significant associations between age groups and adult mental health symptoms. In particular, individuals aged 35 to 49, 50 to 64, 65 to 74, and 75 and older had significantly lower odds of mental health symptoms than individuals in other age groups. These findings are of the most significant importance for understanding the mental health outcomes of the adult study population and have implications for the development of targeted interventions and policies. The significance of these associations suggests that increasing age positively influences adult mental health outcomes. Recent studies by Schönfeld et al. (2017) and Lorem et al. (2017) reported comparable relationships between age and mental health symptoms, which are consistent with our findings. In addition, these studies found lower rates of mental health symptoms among older individuals, indicating the consistency and robustness of the association. The findings suggest that advancing age may protect the mature population from developing mental health symptoms.

In contrast to our findings, Dowdy et al. (2005) found no significant correlation between adult age and mental health symptoms. However, it is essential to note that their study focused on a specific subpopulation and utilized different measurement scales, which may account for the disparate results. Nonetheless, the overall body of evidence, which includes our study and the studies mentioned above, supports the notion that older adults have a reduced risk of mental health symptoms. In addition, gender differences emerged as a significant determinant, with females having a higher incidence of mental health symptoms than males. This result is consistent with Thapa et al. (2020) and Ma et al. (2021), who found higher rates of mental health symptoms among adult females (Nutakor et al., 2023a,b,c). Understanding these gender differences is essential for tailoring mental health interventions and support services to meet the unique requirements and challenges faced by women.

Recent studies have brought to light significant gender discrepancies in mental health outcomes, particularly amongst racial and ethnic minorities (Salk et al., 2017; Fox et al., 2020). For example, research indicates that women often report higher rates of mental health issues than men, a trend that our findings support. This difference can be partly attributed to the unique stressors faced by women, including gender-based discrimination and the simultaneous burden of cultural and societal expectations (Salk et al., 2017; Fox et al., 2020). Additionally, theories such as the differential exposure hypothesis suggest that women may experience a higher frequency and intensity of stressors, including perceived discrimination, which ultimately has a more severe impact on their mental health (Ruglass et al., 2020). It has been observed that coping mechanisms differ between genders, with females possibly relying more on social support (Piccinelli and Wilkinson, 2000). Although social support can be beneficial, it may also increase exposure to stressors in interpersonal relationships. Our study supports this by showing that females in our sample experienced higher rates of mental health issues, which is consistent with previous studies. This highlights the importance of taking gender-specific approaches in mental health interventions, particularly for racial and ethnic minority populations, where gender, culture, and discrimination intersect to add multiple layers of complexity to mental health outcomes.

The study identified several socioeconomic factors associated with adult mental health symptoms. Higher household incomes were associated with a decreased likelihood of mental health symptoms, indicating a positive relationship between income and mental health outcomes. However, disability status, multiple occupation statuses, and long-term unemployment were associated with an increased risk of mental health symptoms. Consistent with recent studies by Reiss et al. (2019) and Henssler et al. (2021) that emphasize the impact of socioeconomic factors on mental health outcomes among adults, these findings demonstrate the influence of socioeconomic factors on adult mental health outcomes. The findings highlight the significance of addressing socioeconomic disparities and supporting vulnerable populations to promote mental health.

There were also significant associations between lifestyle factors and mental health symptoms. Ex-smokers and never-smokers had lower odds of mental health symptoms than current smokers, indicating a positive effect of smoking cessation on mental health. These findings are consistent with those of Xie et al. (2022) and Kock et al. (2023), indicating smoking cessation’s positive effects on adult mental health. Physical activity was also protective against mental health symptoms, with physically active individuals having a lower risk than inactive individuals. Similar findings were reported by McKeon et al. (2022) and Jacob et al. (2020), highlighting the significance of promoting physical activity for adults mental health (Xu et al., 2021).

Table 3 presents the mediation analysis results that cast light on the relationship between adult cultural identity, perceived discrimination, and mental health symptoms. The results suggest that discrimination experiences fully mediate the impact of cultural identity on mental health symptoms. This finding has significant implications for understanding the underlying mechanisms by which cultural identity influences mental health outcomes in this population. Hashemi et al. (2019, 2020) found evidence of the mediating effect of perceived discrimination on mental health disparities among individuals from diverse backgrounds. Understanding the effects of discrimination may inform interventions and policies to reduce discrimination and promote adult mental health.

To better understand the critical role of perceived discrimination in mediating the relationship between cultural identity and mental health symptoms, it is necessary to explore the complex dynamics of this process. Perceived discrimination is a significant stressor that may worsen mental health symptoms by reinforcing negative self-perceptions and increasing psychological distress (Szaflarski and Bauldry, 2019). This indicates that individuals from racial or ethnic minority groups may internalize experiences of discrimination, leading to a heightened sense of vulnerability and isolation, which in turn can have negative impacts on their mental health. The study’s mediation analysis emphasizes the significance of addressing perceived discrimination in interventions that aim to improve mental health outcomes for racial and ethnic minority adults. When professionals and policymakers understand the mechanisms through which cultural identity affects mental health symptoms through perceived discrimination, they can design culturally sensitive interventions that address not only symptoms but also the root causes of mental health disparities. This approach requires a comprehensive understanding of an individual’s cultural background and experiences of discrimination, highlighting the importance of integrated care models that consider the multifaceted nature of mental health in diverse populations.

In terms of policy implications, the findings of this study highlight the need for targeted interventions and policies to address disparities in adult mental health. When devising mental health programs and services, it is crucial to consider racial, ethnic, and socioeconomic factors, as demonstrated by the findings (Alegría et al., 2018; Eylem et al., 2020; Xu et al., 2022). Culturally sensitive approaches, such as providing mental health resources in multiple languages and ensuring that diverse populations are represented in the mental health workforce, may help reduce disparities and enhance mental health outcomes for adult populations (Gopalkrishnan, 2018). In addition, efforts should be made to combat discrimination and encourage social inclusion to enhance the mental health of adults from diverse cultural backgrounds. Implementing community-based mental health programs that cater specifically to the requirements of adults is one of the real-world solutions suggested by the findings (Killaspy et al., 2022). Individuals at higher risk for mental health symptoms, such as those with reduced incomes, disabilities, or discrimination experiences, may receive education, support, and access to mental health services through these programs. Collaborations between healthcare providers, community organisations, and policymakers may facilitate the formulation and implementation of such initiatives. In addition, efforts should be made to increase adult awareness of the mental health benefits of quitting smoking, physical activity, and healthy lifestyle choices (Kumar and Preetha, 2012). Public health campaigns and educational initiatives may empower adults to adopt healthier lifestyles by promoting behavior modification (Kumar and Preetha, 2012).

In our study, we adopt an interdisciplinary approach combining sociology, psychology, and data science knowledge to enhance our analysis of the complex interrelationship between cultural identity, perceived discrimination, and mental health. By utilizing this approach, we can comprehensively understand our research questions, which may be overlooked if we focus on a single discipline. Furthermore, we suggest extending our research to include comparative analysis across diverse geographical and cultural settings. This would allow us to examine the universality or specificity of our findings, providing us with a more profound insight into how cultural context influences the relationship between perceived discrimination and mental health.

Based on the insights we gained from our research, we propose a new intervention that aims to reduce the negative effects of perceived discrimination on mental health. This intervention is based on an interdisciplinary analysis and comparative findings, and it has been designed to be culturally sensitive and adaptable to different community needs. Its objective is not only to address the immediate psychological impacts of discrimination but also to foster resilience and community support among racial and ethnic minority adults. Through this intervention, we aim to illustrate the practical applications of our research, highlighting its potential to contribute to more inclusive and effective mental health support services.

This study has several strengths. First, it is based on the Health Information National Trends Survey (HINTS) 6 data. This study is nationally representative and increases the generalizability of the results to the larger adult population in the United States. The large sample size and rigorous methodology of HINTS 6 provide solid evidence for the identified associations in this study. Second, the study employed binary logistic regression analysis and mediation analysis, enabling a comprehensive examination of the relationships between demographic and lifestyle variables, perceived discrimination, and mental health symptoms. This method provides a nuanced understanding of the complex factors influencing adult mental health outcomes.

Despite these strengths, the study has limitations that must be considered. First, because the data are cross-sectional, it is not easy to establish causal relationships (Nutakor et al., 2023a,b,c). Longitudinal studies would help investigate the temporal relationships between the indicated factors and mental health symptoms. Second, the research employed self-reported measures susceptible to recall and reporting biases. Future research could include objective measures of mental health symptoms and other pertinent variables to strengthen the validity of the findings. In addition, the study was limited to the United States, so the findings may not be directly applicable to other nations or cultural settings. Although our study has found significant associations between demographic factors and mental health symptoms, we acknowledge that these variables could have been included within the mediation analysis to enhance our understanding of the complex ways in which demographic disparities influence mental health outcomes. Therefore, future research should thoroughly examine demographic variables in mediation analyses to understand better the mechanisms driving mental health disparities. By doing so, future studies can contribute to developing more targeted and effective interventions for diverse populations.

## 5 Conclusion

In conclusion, the findings of this study shed light on the significant associations between demographic and lifestyle factors, perceived discrimination, and mental health symptoms among adults. In adult mental health research and interventions, it is essential to consider racial, ethnic, and socioeconomic disparities, cultural identity and discrimination. The study adds to the existing body of knowledge by identifying specific risk and protective factors and highlighting the potential for interventions and policies to enhance mental health in this population. Future research should continue to investigate the intricate interplay of these factors and evaluate the effectiveness of targeted interventions in promoting adult mental health. It is crucial to emphasize the importance of adopting an intersectional perspective when designing public policies and developing mental health interventions. By recognizing and addressing the complex interplay of factors such as racial, ethnic, and socioeconomic disparities, cultural identity, and discrimination, we can work towards achieving more equitable outcomes in mental health care. Embracing intersectionality enables us to move beyond simplistic one-size-fits-all approaches and tailor interventions to individuals’ and communities’ diverse needs and experiences. By focusing on intersectionality in policy formulation and intervention strategies, we can work towards dismantling systemic barriers and promoting mental health equity for all.

## Data availability statement

Publicly available datasets were analyzed in this study. This data can be found here: https://hints.cancer.gov/data/Default.aspx.

## Ethics statement

The studies involving human participants were reviewed and approved through expedited review by the Westat Institutional Review Board, and subsequently deemed exempt by the U.S. National Institutes of Health Office of Human Subjects Research Protections. The studies were conducted in accordance with the local legislation and institutional requirements. The participants provided their written informed consent to participate in this study.

## Author contributions

JN and LZ contributed to conception and design of the study. LZ applied for funding to support this study and supervised the research. JN and EL organized the database. JN, EL, and ND performed the statistical analysis. JN and SA-D wrote the first draft of the manuscript. JN, SA-D, and YC wrote sections of the manuscript. All authors contributed to the article and approved the submitted version.
